# Disease Activity, Functional Ability and Nutritional Status in Patients with Rheumatoid Arthritis: An Observational Study in Greece

**DOI:** 10.31138/mjr.31.4.406

**Published:** 2020-12-28

**Authors:** Anastasia G. Markaki, Konstantinos Gkiouras, Christos Papakitsos, Maria G. Grammatikopoulou, Athena Papatsaraki, Roula Ioannou, Amalia Tsagkari, Theodora Papamitsou, Dimitrios P. Bogdanos

**Affiliations:** 1 Department of Nutrition & Dietetics Sciences, Faculty of Health Sciences, Hellenic Mediterranean University, Sitia, Crete, Greece; 2 Department of Rheumatology and Clinical Immunology, Faculty of Medicine, School of Health Sciences, University of Thessaly, Larissa, Greece; 3 Department of Medicine, School of Health Sciences, Aristotle University of Thessaloniki, Thessaloniki, Greece; 4 Department of Nutrition, KAT General Hospital, Athens, Greece; 5 Health School, Aegean College, Athens, Greece; 6 Department of Histology and Embryology, Department of Medicine, School of Health Sciences, Aristotle University Thessaloniki, Thessaloniki, Greece

**Keywords:** Mediterranean diet, malnutrition, DAS28, nutrition, rheumatoid cachexia, cachexia, functional ability

## Abstract

**Aim:**

The aim of the present pilot study was to assess differences in the nutritional status, Mediterranean diet (MD) adherence, and functional ability among patients with rheumatoid arthritis (RA), according to disease activity.

**Methods:**

A total of 48 patients with RA, outpatients of a hospital in Athens, Greece were recruited. Disease activity was evaluated with DAS28, functional status with the Health Assessment Questionnaire (HAQ), MD adherence with the MedDietScore and malnutrition with the patient-generated subjective global assessment (PG-SGA).

**Results:**

A relationship was noted between DAS28 and HAQ, indicating a reduced functional status with increased RA activity. Although MD adherence differed between DAS28 categories, no specific differences were noted in the PG-SGA or the MedDietScore in the post-hoc analyses. According to the PG-SGA, no need for nutritional intervention was noted among participants.

**Conclusions:**

The origin of the participants might have reduced the differences between MD adherence and DAS28. In parallel, the PG-SGA does not appear sensitive in detecting muscle-related malnutrition among patients with RA.

## INTRODUCTION

Rheumatoid arthritis (RA) is a chronic condition, with patients receiving complex and diverse medications, often associated with adverse events.^1–3^ As a result, research interest on possible lifestyle changes with fewer adverse events has mushroomed recently, with a plethora of diet regimes and oral nutrient supplements (ONS) being investigated.^4–7^

In particular, studies using specific diets, such as the Mediterranean dietary pattern, have shown positive findings in tampering down pain and reducing the number of swollen joints.^6,8^ Individual components of the Mediterranean diet (MD) including poly-unsaturated fatty acids (PUFA), when provided as ONS, have been shown to reduce inflammation and the progression to pharmacotherapy.^6^ In parallel, according to a recent meta-analysis,^9^ 15 to 32% of patients with RA experience rheumatoid cachexia, as a result of joint destruction, subsequent muscle inactivity,^10^ the high levels of sarcoactive inflammatory cytokines – including the tumour necrosis factor α (TNF)-α and interleukin (IL) 1β,^11^ and due to the systemic inflammatory process experienced in RA.^12^ In addition, factors used commonly in RA to tamper down inflammation, including TNF inhibitors and the IL 6 receptor blocker, further aggregate body protein and energy metabolism, inducing body composition alterations.^13^ Although important, relevant data from Greece are limited.

The aim of the present pilot study was to assess differences in the nutritional status, MD adherence and functional ability among patients with RA, according to disease activity.

## METHODOLOGY

### Participants and research question

RA participants were recruited from consecutive outpatients visiting the Rheumatology clinic situated at the KAT General hospital in Athens, Greece, during 2014 (from April, to December of the same year). Patient characteristics are presented in **Table 1**. Eligible patients: 1) were adults (exceeding 18 years of age) on the basis of a definite disease (RA) irrespectively of whether their serological status (rheumatoid factor and anti-cyclic citrullinated peptide antibodies) was known or not to the treating physicians, 2) could communicate efficiently in the Greek language and 3) and provided participation consent. Permission for the study was granted by the Hellenic Mediterranean University and the KAT General Hospital. All patients provided informed consent prior to their participation. The participating institutions (Hellenic Mediterranean University and KAT General Hospital) have obtained an Ethics Committee approval.

**Table 1.** Description of study variables and tests for difference according to sex.

**Characteristic**	**Total sample (*n*=48)**	**Men (*n*=8)**	**Women (*n*=40)**	**P-value**
Age (years)	60.29 ± 12.94	60.13 ± 12.82	60.33 ± 13.13	0.969^§^
BMI (kg/m^2^)	25.67 (23.28–30.13)	26.06 (23.95–28.96)	25.31 (22.72–30.28)	0.611^¥^
BMI categories^14^				
*Normoweight*	22 (45.83%)	3 (37.50%)	19 (47.50%)	0.884^£^
*Overweight*	14 (29.17%)	3 (37.50%)	11 (27.50%)	
*Obese*	12 (25.00%)	2 (25.00%)	10 (25.00%)	
Disease duration (months)	63.00 (33.00–120.00)	56.00 (41.50–109.5)	73.50 (28.50–120.00)	0.897^¥^
MedDietScore	30.00 (27.00–31.50)	29.00 (28.00–30.50)	29.55±3.84	0.377^¥^
HAQ	0.54 (0.11–0.95)	0.09 (0.01–0.40)	0.65±0.44	0.037^¥^
PG-SGA score	0.50 (0.50–2.00)	0.50 (0.00–1.25)	0.50 (0.50–2.00)	0.193^¥^
PG-SGA categories				
*<2*	32 (66.67%)	6 (75.00%)	26 (65.00%)	1.000^£^
*2 to <4*	11 (22.92%)	2 (25.00%)	9 (22.50%)	
*4 to <9*	4 (8.33%)	0 (0.00%)	4 (10.00%)	
*≥9*	1 (2.08%)	0 (0.00%)	1 (2.50%)	
DAS28 score	4.42 (2.74–6.24)	4.08 ± 1.74	4.55 ± 2.08	0.553^§^
CRP (mg/dL)	0.80 (0.36–3.57)^1^	2.58 ± 2.40^1^	0.80 (0.38–3.30)	0.855^¥^
Albumin (g/dL)	3.90 (3.65–4.00)^2^	3.96 ± 0.59^1^	3.90 (3.65–4.00)^3^	0.752^¥^
ESR (mm/h)	36.50 (15.50–55.00)	44.25 ± 23.66	34.50 (15.00–55.00)	0.466^¥^
Haemoglobin (g/dL)	12.39 ± 1.55	13.02 ± 2.07	12.26 ± 1.43	0.211^§^

BMI: body mass index; CRP: C-reactive protein; DAS28: Disease Activity Score-28;^15^ ESR: erythrocyte sedimentation rate; HAQ: Health Assessment Questionnaire;^19^ MedDietScore: Mediterranean diet score;^22^ PG-SGA: Patient-Generated Subjective Global Assessment.^21^

Values are means ± standard deviations or medians (with 25^th^ – 75^th^ percentile) for continuous variables or frequencies (with percentages) for categorical variables.

§ refers to independent t-tests;

¥ refers to Mann-Whitney tests;

£ refers to Chi-square tests,

1 1 missing value;

2 5 missing values;

3 4 missing values.

### Research question and design

The research question of this study in the format of P.E.O. (Population, Exposure and Outcomes) was addressed as follows: In patients suffering from RA (P), do different disease activity levels (E) associate with differences in their nutritional or functional status (O)?

The study had a cross-sectional research design, with all measurements of exposure and outcome variables been recorded once for each patient (either measured by the authors of the study, or extracted from the patients’ medical records).

### Anthropometry

Body weight and height of participants were recorded with a Seca 711 mechanical scale (Seca, Hamburg, Germany) and a Harpenden wall-mounted stadiometer, respectively (Holtain Limited, Crosswell, Crymych, Pembs, UK). For each patient, the body mass index (BMI) was calculated as body mass (kg) divided by stature (m^2^). Weight status was defined according to the World Health Organization^14^ BMI cutoffs for normoweight (18.5 < BMI ≤ 24.99 kg/m^2^), overweight (25 < BMI ≤ 29.99 kg/m^2^) and obesity (≥ 30 kg/m^2^).

### Disease activity and functional status

The Disease Activity Score using 28 joint counts (DAS28)^15^ was used based on the erythrocyte sedimentation rate (ESR) of participants was selected. It is based on the swollen joint counts (SJCs) and the tender joint counts (TJCs). Scores <2.4 represent remission, low (≤3.2), moderate (≤5.1) or severe (>5.1) disease activity, respectively.^16,17^

C-reactive protein (CRP), albumin and haemoglobin levels were recorded for each patient from their medical records. Disease duration was also recorded from medical records and was timed since the diagnosis of each patient (expressed in months).

Functional status/disability of participants was evaluated using a validated translation of the Stanford Health Assessment Questionnaire (HAQ) in the Greek language.^18,19^ The HAQ offers a patient-oriented outcome assessment of eight functional domains based on 20 specific functions, evaluating patient difficulty in performing daily activities during a recall period of one week.^19,20^

### Malnutrition and diet quality

Malnutrition was assessed with the Patient-Generated Subjective Global Assessment (PG-SGA).^21^ The PG-SGA provides a numerical score rating nourishment of patients. Higher scores are indicative of increased malnutrition risk whereas, a score ≥ 9 indicates an urgent need for nutrition intervention. In particular, the scores allow for triaging nutrition interventions, including symptoms management, the need for nutrition education for both the patient and the family, as well as specific dietary manipulations including (1) the need for enteral or parenteral nutrition, (2) the provision of additional or larger food portions to meet energy requirements, or (3) a need for oral nutrition supplements (ONS) to meet micronutrient needs.^21^

The Mediterranean diet adherence was assessed via the MedDietScore score for each patient as previously proposed.^22^ The MedDietScore assesses the frequency of consumption of eleven distinct foods/food groups, including fish, non-refined cereals, fruits, vegetables, olive oil, alcohol, poultry, red meat, full-fat dairy, potatoes and legumes. The score ranges between 0 to 55, with greater scores being indicative of greater adherence to the Mediterranean diet.

### Statistical analysis

Data are reported as means along with their standard deviations (SDs) for normally distributed variables, or as medians alongside their corresponding 25th and 75th percentiles when non-normally distributed variables were concerned. Deviations from the normality assumption were assessed with Shapiro-Wlik’s test. Independent t-tests assessed differences in normally distributed variables and the assumption of homoscedasticity was tested with Levene’s test. Categorical variables are presented as frequencies with their corresponding percentages and were assessed with the Chi-square test. Mann-Whitney tests assessed differences in non-normally distributed samples. For comparing nutritional (BMI, MedDietScore, and PG-SGA) and health assessment (HAQ) among the DAS28 categories, the Kruskal-Wallis tests were performed due to lack of normality on ANOVA’s (Analysis of Variance) residuals and/or lack of homogeneity of variances. Post-hoc comparisons were performed with the Bonferroni correction. Correlations among DAS28 as a continuous variable and BMI, MedDietScore, PG-SGA, and HAQ were assessed with Spearman’s rho correlation coefficient. SPSS version 25 (SPSS, Chicago, IL, USA) was used for the analyses and the level of significance was set at 0.05.

## RESULTS

The sample was consisted of 48 RA patients with a mean age of 60.3 ± 12.9 years old. Most subjects were normoweight (45.8%) with a median BMI of 25.7 kg/m^2^ and a median MedDietScore of 30 (**Table 1**). No differences were noted between men and women, except for an increased HAQ score for the latter (p=0.037).

### Associations of DAS28 with nutritional and general health variables

DAS28 categories differed on HAQ score (p=0.027) (**Table 2**). Specifically, in post-hoc comparisons, only participants in remission (DAS28 ≤2.6) compared to participants with moderate DAS28 scores (2.6 to 3.2) had lower HAQ score (p=0.015). Additionally, DAS28 (as a continuous variable) demonstrated a week, positive correlation with HAQ (rho=0.296, p=0.041).

**Table 2.** Differences according to the DAS28 categories.

	**Disease activity based on DAS28**				
**Variable**	**Remission** [DAS28 < 2.6] (*n*=11)	**Low** [DAS28 ≤ 3.2] (*n*=5)	**Moderate** [DAS28 ≤ 5.1] (*n*=12)	**High** [DAS28 >5.1] (*n*=20)	**P-Value**
**BMI (kg/m^2^)**	28.57 ± 7.04	24.98 (23.88–25.87)	24.34 (22.40–28.89)	26.40 ± 3.91	0.853
**MedDietScore**	27.82 ± 4.21	27.00 (27.00–29.00)	31.83 ± 2.72	29.3 ± 3.37	0.037
**HAQ score**	0.15 (0.04–0.43)	0.58 ± 0.64	0.86 ± 0.32	0.61 ± 0.49	0.027
**PG-SGA**	0.50 (0.50–0.50)	1.30 ± 1.20	0.50 (0.00–0.75)	1.50 (0.50–3.00)	0.166

BMI: body mass index; DAS28: Disease Activity Score-28;^15^ HAQ; Health Assessment Questionnaire;^19^ MedDietScore: Mediterranean diet score,^22^ PG-SGA: Patient-Generated Subjective Global Assessment.^21^

Values are means ± standard deviations or medians (with 25th – 75th percentile).

Although the Kruskal-Wallis test indicated the presence of at least one difference among the DAS28 categories on MedDietScore (p=0.037) (**Table 2**), all post-hoc tests remained non-significant (all p>0.05) indicating that no specific DAS28 categories were significantly different from one another. Furthermore, DAS28 (as a continuous variable) did not demonstrate a correlation to the MedDietScore (rho=0.016, p=0.916). No further significant differences or correlations were noted for BMI or PG-SGA.

## DISCUSSION

The present pilot study indicates the existence of a relationship between disease activity and disability among patients with RA. In parallel, disease activity was associated with various levels of MD adherence. Malnutrition assessment failed to associate with disease activity.

Although small in sample size, the current findings are in agreement to previous research indicating a relationship between HAQ and DAS28.^23–25^ Boyd^23^ suggested that this tight connection is stronger at diagnosis (first visit), with a tendency to weaken as time passes after the diagnosis. On the other hand, age of participants did not appear to induce changes in this association,^23^ although it has been suggested that in older patients, age-related factors might act synergistically in tampering down functional status further. Previous research has showed that increased disease activity, older age, and female sex consist of HAQ predictors, irrespectively of the DAS28 score.^26^ In the present analysis, disease duration was long, whereas on the other hand, the majority of participants consisted of women of older age, and these factors might have contributed to weakening the DAS28-HAQ association. Nevertheless, the existence of a connection was noted, indicating that elevated increased RA activity and joint destruction was associated with deteriorated functional capacity. According to Drossaers-Bakker,^25^ disease activity is in fact the most pivotal determinant of the HAQ score and both should be evaluated at every patient visit to the record course of the disease.

In a recent review, Vranou and associates^5^ suggested that the quality of evidence linking the MD to RA outcomes appears to be of low evidence, stemming mainly from observational studies. Similar issues were also suggested in a systematic review and meta-analysis by Forsyth and collegues.^8^ Herein, although differences were noted in the MedDietScore between distinct DAS28 tiers, post-hoc analyses failed to indicate significant differences. It can also be argued that the finding of non-statistically significant differences in the compared groups cannot rule out the possible existence of clinical differences since patients with moderate activity demonstrated the higher MedDietScore. Moreover, given that the sample derived from a Mediterranean country where the MD consists of the main diet prototype adhered, it is possible that most of the participants demonstrated an adequate degree of MD adherence limiting between-group differences.

No differences were noted in the malnutrition score in distinct DAS28 categories. Although nutrition appears to be an important component of RA initiation and progression,^5,27^ BMI is not sensitive enough to detect malnutrition,^28^ with none of the participants herein being underweight according to their BMI. On the other hand, muscle protein degradation has been shown to occur in RA,^10,29,30^ and hand grip strength, skinfold or body composition measurements might have produced more accurate results as compared to the PG-SGA. Rheumatoid cachexia has been suggested to correlate with disease activity and disability,^10^ further complicating outcomes and health status of patients. The PG-SGA used herein did not appear sensitive enough to depict malnutrition, as the only anthropometric index used to calculate the PG-SGA is body weight. Furthermore, all participants used in the present study were outpatients and it is likely that the inclusion of in-ward patients^28^ might have altered the malnutrition level of the sample. Nevertheless, according to the PG-SGA, no specific triage recommendations were required for the participants.

Limitations of the present study include its small sample and cross-sectional design for demonstrating a causal relationship and further limit the generalizability of the findings. In parallel, if the DAS28-CRP was used instead of the DAS28-ESR, results might have differed.^31^ Finally, the small sample size and the lack of funding prohibited additional analyses adjusted for potential confounders such as socio-demographic characteristics, occupation, comorbidities and lifestyle parameters.

The results indicate that disease activity and functional disability are aligned in RA. Given the design of the study, it is difficult to understand the role of the MD, however, most patients appear to adhere well to the MD prototype, either due to origin, or health consciousness. Further studies are needed to understand the disability and nutritional status of RA patients in Greece, as data are limited.
